# A Systematic Review of Vitamin D during Pregnancy and Postnatally and Symptoms of Depression in the Antenatal and Postpartum Period from Randomized Controlled Trials and Observational Studies

**DOI:** 10.3390/nu14112300

**Published:** 2022-05-30

**Authors:** Jacqueline F. Gould, Robert A. Gibson, Tim J. Green, Maria Makrides

**Affiliations:** 1 SAHMRI Women and Kids, South Australian Health and Medical Research Institute, 72 King William Road, North Adelaide, SA 5006, School of Psychology and the Discipline of Paediatrics, Faculty of Health and Medical Sciences, The University of Adelaide, North Terrace, Adelaide, SA 5000, Australia; 2 School of Agriculture, Food and Wine, The University of Adelaide, North Terrace, Adelaide, SA 5000, SAHMRI Women and Kids, South Australian Health and Medical Research Institute, 72 King William Road, North Adelaide, SA 5006, Australia; robert.gibson@adelaide.edu.au; 3 SAHMRI Women and Kids, South Australian Health and Medical Research Institute, 72 King William Road, North Adelaide, SA 5006, School of Medicine, Faculty of Health and Medical Sciences, The University of Adelaide, North Terrace, Adelaide, SA 5000, Australia; tim.green@sahmri.com; 4 SAHMRI Women and Kids, South Australian Health and Medical Research Institute, 72 King William Road, North Adelaide, SA 5006, School of Medicine, Discipline of Paediatrics, Faculty of Health and Medical Sciences, The University of Adelaide, North Terrace, Adelaide, SA 5000, Australia; maria.makrides@sahmri.com

**Keywords:** vitamin D, vitamin D deficiency, supplementation, depression, pregnancy, postpartum, systematic review

## Abstract

Depression is a common mood disorder associated with childbirth and is hypothesized to be affected by low vitamin D. This systematic review identified two randomized controlled trials (RCT) of vitamin D supplementation for the treatment or prevention of depressive symptoms in the perinatal period, as well as 18 observational studies of vitamin D exposure and depression in the antenatal and postnatal periods. Both RCTs claimed an improvement in depressive symptoms in the vitamin D group, although the sample sizes were too small to draw firm conclusions. The case-control and cohort studies had mixed findings and were limited by study quality. There were inconsistent results within the few studies with a more robust methodology or within samples restricted to women likely to have depression. The current evidence is inconclusive due to the poor quality and heterogeneity of studies, likely contributing to the contradictory findings. Given there are already numerous RCTs of prenatal vitamin D supplementation, we recommend adding an appropriate measure of depression in the perinatal period to assist in resolving the uncertainty.

## 1. Introduction

Postpartum depression (PPD) is common, with 19% of mothers experiencing depression within 12 weeks of birth and another 10–20% of women experiencing PPD within the first year [1,2], and for 8% of women symptoms persist beyond a year [3]. Women with depression in the perinatal period may experience mood disturbances (including sadness, loss of pleasure, guilt, or worthlessness), sleep disturbances (unrelated to their pregnancy or infant), appetite disturbances, weight loss, and suicidal ideation. PPD has adverse implications for mother-infant attachment and child development [4,5,6].

The prevalence of depression in the perinatal period is similar across race, parity, age, education, and socioeconomic status [1] and there is no clear cause [7,8]. Consequently, it is not possible to accurately predict which mothers will develop depression or determine how best to prevent PPD in the perinatal period. However, some evidence suggests that low vitamin D may increase the risk of mood disorders such as PPD [9,10,11].

Vitamin D is obtained when the skin is exposed to sunlight, and to a lesser extent from diet. Vitamin D, from diet or skin synthesis, is then hydroxylated to 25-hydroxyvitamin D (25OHD), the major circulating form of vitamin D and the best indicator of vitamin D status [12]. To be fully activated, 25OHD must undergo a second hydroxylation to 1,25-dihydroxyvitamin D. This active form is a nuclear steroid that binds to the vitamin D receptors [13] that are present in many tissues, including the human brain [14], providing biological plausibility for a role in neurological functioning. Initial suggestions that vitamin D plays a role in mood disorders arose from seasonal affective disorder, a mood disorder with symptoms of depression that occurs in the winter months, where vitamin D synthesis by sunlight is low [15]. Meta-analyses of observational studies have reported an association between low 25OHD and mood disorders such as depression [9,10]. The meta-analysis of trials of vitamin D supplementation and depression suggests some benefit, although the results are variable, and in most instances, the study quality is poor [16]. Vitamin D supplementation is not currently recommended for the treatment of depressive symptoms due to the low quality of the evidence; however, depression in the perinatal period is absent from this literature [16].

Pregnancy and lactation may be a demanding time in terms of nutrient requirements, where maternal nutrient reserves may become depleted to ensure adequate nutrition for the developing baby [17]. Increased prevalence of poor vitamin D status has been reported in pregnant women, based on low 25OHD, in many populations globally [18,19]. Suboptimal vitamin D status during pregnancy or postpartum may contribute to symptoms of depression. If effective, ensuring women have sufficient vitamin D may be a simple, safe, and cheap method of preventing, or reducing symptoms of depression in the perinatal period [20,21]. However, reviews of vitamin D in the perinatal period have reported conflicting inconclusive results [22,23,24,25,26], and have not actively included depression as an outcome of interest [18,27], or have not included all concurrently published studies [11,26], and to date all reviews have been limited to observational studies [24,25,26]. We aim to conduct the first systematic review of trials and observational studies of vitamin D and depression during the perinatal period and postpartum. We will determine whether there is consistency between the recently published trials capable of providing causal evidence for a role of vitamin D in antenatal and postnatal depression and the body of observational evidence.

## 2. Materials and Methods

We conducted our systematic review according to the guidelines outlined in the Preferred Reporting Items for Systematic Reviews and Meta-Analysis [28]. This systematic review is registered on the PROSPERO registry (https://www.crd.york.ac.uk/prospero/; ID CRD42022328361, last edited on 10 May 2022).

Published articles were eligible for inclusion in this review if they were a trial of vitamin D supplementation during the antenatal or postpartum period, or if they were an observational study of vitamin D (25OHD) status, vitamin D intake, or vitamin D exposure), and the study included a measure of depressive symptoms (such as clinical diagnoses of a depressive disorder, use of medication for a depressive disorder in the perinatal period, and questionnaires measuring depressive symptoms). Animal studies and manuscripts not published in English were excluded.

We searched PubMed for eligible articles, with weekly search alerts to capture new potentially eligible publications for inclusion up until October 2021. Reference lists of eligible articles, as well as any similar reviews were also screened for relevant manuscripts. Titles of articles were screened, followed by abstracts and full text where needed to determine eligibility.

The included studies were reviewed, and pertinent information was summarized in tables. Information of interest included descriptions of the study design (trial, case-control, and cohort), sample population (characteristics, size of sample, inclusion, and exclusion criteria), intervention details (timing, dose, and duration of any vitamin D supplements, and inclusion of other nutrients) or details of vitamin D exposure (such as timing of measurement, analytical method, definition of status, and definition of deficiency, if any), timing and measurement of depressive symptoms, as well as results. We also noted limitations and possible bias, such as small sample, suboptimal exposure, or outcome measures, or other indications of poor study quality (for example, inadequate consideration of confounders).

Trials of vitamin D supplementation were considered separately to observational studies. Observational studies were subdivided into case-control studies or cohort studies, and results of explorations in clinical samples are discussed separately from general (non-clinical) samples. Given the growing concerns around the effects of insufficient vitamin D, we considered the results of analyses of vitamin D status as a continuous variabl, and categorical variable separately, with an emphasis on explorations of deficient or insufficient vitamin D status. Where unadjusted and adjusted analyses are reported, only the fully adjusted results are considered here.

## 3. Results

**Figure 1:**
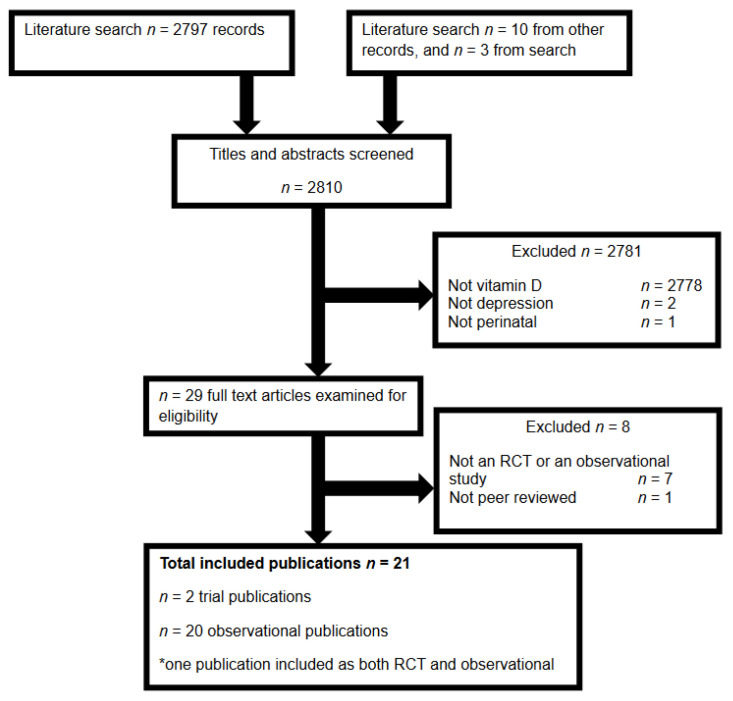
Flow of publications through the literature search and screening for eligibility.

Our search identified a total of 2810 manuscripts, of which there were 21 eligible articles (see Figure 1 for flow); two RCTs [29,30]; and 18 observational studies [29,31,32,33,34,35,36,37,38,39,40,41,42,43,44,45,46,47,48,49], including one trial that also reported associations between depression and vitamin D status at trial entry [29] and two cohorts that reported results across multiple publications [42,43,47,48]. One potentially eligible case-control study was excluded, as it was reported as a dissertation from 2013 and had not been peer reviewed [50].

### 3.1. Trials of Vitamin D and Depression in the Perinatal Period

Although we identified many perinatal vitamin D RCTs, only two assessed symptoms of depression and both were conducted in Iran, (Table 1) [29,30].

#### 3.1.1. Trial Design, Randomization, and Blinding

The two vitamin D trials identified were described as double-blind RCTs, one conducted during pregnancy and the other postpartum [29,30]. No primary outcome was specified in the prenatal trial [29] and the postnatal trial had joint primary outcomes of PPD and serum concentrations of 25OHD [30]. Both trials included a control group that was given a placebo [29,30]. One trial involved two randomization groups [29] and the other involved three randomization groups [30], to which women were randomized via block design, although specific details regarding randomization techniques were not reported in either trial. Blinding information was absent in the report for the prenatal trial, apart from stating it was single-blinded, and women likely knew their group allocation [29]. Women and staff in the postnatal trial were likely blinded through use of two-digit identification numbers to identify participants and supplement bottles (packed by a non-study staff) [30].

#### 3.1.2. Trial Sample Details

The trials differed in their target sample and recruitment strategy, although both were conducted in small samples of Iranian mothers with less than 100 per group [29,30]. Pregnant women approached at a prenatal hospital clinic were excluded if they had depression or were likely to have depression [29]. The postnatal trial recruited a sample of women from a psychiatric outpatient clinic if they had depressive symptoms but were not using antidepressants [30]. Women were excluded if they had sufficient vitamin D status [30].

#### 3.1.3. Vitamin D Interventions

Both trials randomized women to oral vitamin D supplements or a placebo [29,30]. Pregnant women were provided with 50 µg of vitamin D/3 from two tablets per day throughout the last trimester of pregnancy [29], or received 1250 µg vitamin D3 as fortnightly supplements postpartum (exact timing not reported) over an 8-week period [30]. The postpartum intervention had three randomization groups, one of which also received calcium [30], whilst women in the prenatal trial may have been taking a regular multivitamin containing vitamin D [29].

#### 3.1.4. Depression Outcome Measure and Timing in Trials

Both trials measured depression using the Iranian version of the Edinburgh Postnatal Depression Scale (EPDS). The EPDS is an appropriate, widely used questionnaire that can screen for depressive symptoms specifically in the postpartum period with high sensitivity (68–95%) and high specificity (78–96%) against a clinical psychiatric diagnosis of PPD [51,52]. It is typically self-completed and a score of more than 12 is most commonly used to indicate a probable depressive disorder [53,54]. In both trials, the EPDS was administered through an interview with study staff, and women were considered at risk of depression if they scored >13 [29,30]. In the non-depressed sample, women completed the EPDS three times, once during pregnancy and twice postpartum [29], whilst women in the depressed sample completed the EPDS at the end of the intervention (8 weeks after enrolment) [30].

#### 3.1.5. Trial Efficacy

Vitamin D supplementation in both trials resulted in a decrease in EPDS scores in the treatment group(s) compared with the control group [29,30]. In the non-depressed population, depression scores also decreased in the control group, however, not to the same extent as the vitamin D group [29].

### 3.2. Observational Studies of Vitamin D and Depression in the Perinatal Period

There were 21 publications with an exploration of vitamin D and depression in the perinatal period (summarized in Table 2) [29,31,32,33,34,35,36,37,38,39,40,41,42,43,44,45,46,47,48,49]. Two cohorts reported outcomes across more than one manuscript [42,43,47,48]. One RCT of vitamin D also reported the association between depressive symptoms and vitamin D status and has been included as an observational study (Table 2) as well as a trial (Table 1) [29].

#### 3.2.1. Observation Study Designs

None of the observational studies appeared to be designed and conducted specifically to assess the association between vitamin D and symptoms of depression in the perinatal period. Most were established as general pregnancy cohorts [31,34,35,41,42,47,49]. One was a prenatal vitamin D RCT [29], one was a convenience sample of a larger study that appeared to be a vitamin D dose-response trial [33], and two studies were originally prenatal omega-3 RCTs [37,40]. Three studies were case-control studies that appropriately compared the vitamin D status of women classified as depressed to women not classified as depressed [34,44,45], and three cohort studies analyzed their sample as though they conducted a case-control study [32,36,49].

#### 3.2.2. Observation Study Recruitment and Sample Details

Most studies were conducted in high income countries such as the United States [31,33,39,40,43,48,49], Australia [35,37,38], Denmark [34], The Netherlands [41], and Japan [42,47]. There were a few studies in Iran [29], India [44], China [32], and Turkey [36,46]. Most studies recruited women presenting at antenatal clinics [29,37,39,40,41,42,43,46,47,48,49], whilst others recruited women in hospital after birth [32] or from postpartum or public health clinics [44,45]. Two studies did not specify how or when women were originally recruited [33,34] and another did not recruit women, but accessed medical records for all births in a specific region and time [38]. Sample sizes of the original studies ranged from 126 to 91,000 women, but most had <300 women. Inclusion and exclusion criteria varied and were not clear in some studies [36,41,42,46].

##### Women with PPD

All case-control studies targeted clinical samples as cases [34,44,45]. The largest was originally a national birth cohort of 91,000 women, where cases were women who filled a prescription for an anti-depressive medication within the first year of birth women who had not filled a prescription were controls [34]. Women were classified in another study according to EPDS score (≥10 = cases, <10 = controls) at the study entry, although women were excluded if they appeared to have depression [44]. In the smallest study, cases were not defined, but appeared to be based on a depression questionnaire at enrolment [45].

One cohort only included women who appeared to be at-risk of depression but excluded women with current depressive disorder or anti-depressant medication use [40]. Of the three cohort studies that analyzed their sample as case-controls, two defined cases using EPDS score (≥12 = cases) but excluded women at risk of developing PPD or who underwent prenatal psychiatric care [32,36]. The third study based the definition of cases on a screening tool of depressive symptoms [49]. In all three studies that were not true case-control studies by design, women without PPD (considered as controls) were not matched to cases [32,36,49].

##### Women without PPD

There were 12 studies conducted in general populations [29,31,33,35,37,38,39,41,42,43,46,47,48], half of which actively excluded women if they were under psychiatric care during their pregnancy or had psychiatric illness [29,39,43,46,48], had a history of depression [43], or if the women had risk factors for developing depression or their infant was admitted to a neonatal intensive care unit [29,45].

#### 3.2.3. Observation Study Vitamin D Exposure Assessment

25OHD in blood is considered to be the most relevant indicator of vitamin D status. Liquid chromatography-tandem mass spectroscopy (LC-MS/MS) [55] is becoming increasingly employed to measure nutrient status in blood and this was the reported method of five studies [31,34,35,37,39]. One study claimed to have analyzed vitamin D metabolite ratio using LC-MS/MS [49] and other mechanisms included rapid direct radioimmunoassay [33,40], chemiluminescence immunoassay [29,43,48], enzyme-linked immunosorbent assay [36,44,45], enzyme immunoassay [41], high performance liquid chromatography [46], and E601 modular analyzer [32]. One of the larger studies did not specify how 25OHD status was measured [38]. Diet is a poor indicator of vitamin D as sunlight is generally the primary source, yet in a Japanese study dietary intake (of fish and eggs but not vitamin D supplements) was used to define vitamin D exposure [42,47].

Blood 25OHD status was measured during early pregnancy [31,38,39,40,43,48], or mid-late pregnancy [29,34,35,36,46,49]. Two studies used cord blood samples [37,39] and another took a fasting maternal blood sample 24–48 h after birth [32]. In most studies, the measure of vitamin D status was prospective, prior to the assessment of depressive symptoms [32,34,35,36,37,40,46,47,48], although some measured both the exposure and outcome simultaneously [29,31,38,40,42,44] and two did not clarify the timing of exposure relative to the outcome measure [43,45]. 25OHD status was quantified differently (nmol/L, nmol/L) in the included studies and of those that explored deficient or insufficient vitamin D, definitions varied [29,32,33,34,35,36,37,39,40,43,44,45,46,48].

#### 3.2.4. Observation Study Depression Outcome Measure and Timing

Clinical diagnosis is the most robust and accurate indication of depression; however, only one study used a tool reportedly capable of diagnosing depressive disorder: the Mini International Neuropsychiatric Interview (MINI) [40]. The MINI is an interview for major depressive disorder and anxiety symptoms, as well as generalized anxiety disorder. Another study defined cases and control based on the use of antidepressant medication [34], which is reasonably robust but would reflect serious depressive disorder and would miss women diagnosed as depressed but not willing to take medication in the perinatal period.

For research purposes, depression is often measured through brief self-completed questionnaires that screen for symptoms. The EPDS was specifically developed to measure symptoms in the postpartum period whilst accounting for common postpartum difficulties such as sleep, but is not recommended for use within 14 days of delivery [52]. The EPDS was the most commonly used tool in the included studies [29,32,33,35,36,37,38,39,44,46,47,48], although two studies inappropriately administered it within one week of birth [35,36], and one of these studies used an abbreviated, unvalidated version [35]. Cut-offs for categorization of PPD varied between studies, from >9 to ≥13.

Several other screening questionnaires were administered, although they were not necessarily designed or validated for use in the perinatal period. The Beck Depression Inventory (BDI) is a 21-item scale of depressive symptomatology that has had minimal validation for use in pregnant and postpartum women. The BDI was administered in two studies and neither specified the cut-off score use to define depression [40,45]. The Center for Epidemiological Studies Depression scale (CES-D) is another 21-item scale not adapted for use in the perinatal period, and is reported to have 60% sensitivity for detecting PPD, hence missing a large proportion of patients with PPD [56]. The CES-D was administered in four studies that all defined depression as a score ≥16 [41,42,43,49]. The Depression, Anxiety, and Stress Scale (DASS) and the Patient Health Questionnaire Depression Module (PHQ) were simultaneously administered in one study [31], although neither are well adapted to pregnancy and the DASS is considered a measure of stress and anxiety as well as depression. All depression screens used were developed in English-speaking Western samples but were translated into Chinese [32], Tamil (India) [44], Turkish [36,41,46], Iranian [29,45], Dutch [41], Arabic [41], and Japanese [42] in the included studies. Furthermore, these screening questionnaires are typically designed to be self-completed directly by the participant but were administered via interview in some studies, which may have influenced the responses [29,45].

Eight studies explored depression during pregnancy [29,31,38,39,40,41,42,43], and 14 studies targeted PPD [32,33,34,35,36,37,39,40,44,45,46,47,48,49], with several cohorts measuring symptoms in both the antenatal [39,42,43] and the postpartum periods [39,40,42,47,48]. In one study, symptoms were measured shortly after birth and likely were more reflective of depression in pregnancy [35].

#### 3.2.5. Observation Study Consideration of Confounders

The most important confounder to consider when exploring vitamin D is that season as sun exposure is the main source of vitamin D. Several studies took season into account by including it as a potential confounder in statistical models [31,33,35,38,40,41,43], but there is only one standardized 25OHD status according to the season at the time of blood draw, which is the most robust method of accounting for the season [37]. Other key confounders that should be considered as a minimum for a study of vitamin D and depression include maternal age, education (and/or other indicators of socio-economic status), history of depression, and multivitamin or vitamin D supplement use. The minimum confounders were accounted for in the statistical models of only one study [37]. Season was absent in more than half of the studies [29,32,34,36,39,42,44,45,46,47,49]. Most studies included some but not all key confounders in their models but with no consistency; one study did not adjust for any key confounders [46], and two studies did not report adjusting for any confounding factors [29,36].

#### 3.2.6. Observation Study Results

##### Women Considered to Have Depression

Among the studies conducted within samples that were classified as depressed, three case-control studies had different conclusions [34,44,45], as did three studies that analyzed their cohort as a case-control study [32,36,49]. The largest case-control study found that deficient 25OHD status did not increase the likelihood of being a case, but unexpectedly reported an increased likelihood of having PPD when 25OHD status fell into one of the higher categories (>80 nmol/L) [34]. In contrast, the other smaller case-control studies found that cases were more likely to be categorized as deficient [44,45], and have lower mean 25OHD status compared with controls [45].

The three cohorts that compared the 25OHD status of women characterized as depressed and not depressed all found that vitamin D levels were lower in depressed women [32,36,49] and both studies that explored 25(OHD deficiency found that depressed women were more likely to be vitamin D deficient than non-depressed women [32,36].

A small cohort restricted to women at risk of depression detected no statistically significant increase in depressive symptoms with continuous 25OHD status, or deficient vitamin D [40].

##### General Samples

Of the studies in general non-clinical samples, there were eight explorations of continuous 25OHD levels [29,31,37,39,40,41,43,46,48], and nine explorations for deficient or insufficient 25OHD status [33,37,38,39,40,41,42,43,46,47], and a further two that split their sample into quartiles based on 25OHD [31,35]. Two studies with continuous 25OHD identified an increased risk of depressive symptoms as 25OHD decreased [39,41], four found no association [29,31,37,46], and one cohort reported both a negative association and no association depending on the timing of the outcome measure [43,48]. Five studies found 25OHD deficiency or insufficiency increased the risk of having depression [33,38,39,41,43], one found no increased risk (although all women were taking vitamin D supplements during pregnancy) [46], and one found conflicting results depending on whether women were randomized to omega-3 supplements or a placebo and the timing of the outcome measure [37]. PPD at 6 months was not associated with deficient 25OHD and at 6 weeks postpartum there was an increased risk of PPD in deficient women if they received the placebo [37]. Two studies that split the sample according to quartiles of 25OHD levels conversely found no association between vitamin D status and depression [31] and that the lowest quartile (25OHD < 47 nmol/L) had an increased risk of PPD [35].

Higher dietary intake of vitamin D in pregnancy was associated with lower risk of concurrent depressive symptoms [42], but not subsequent PPD [47].

##### Results according to Key Quality Indicators

When considering the results of the studies that assessed 25OHD using LC-MS/MS, two found no association with depression [31,34], two found a negative association between 25OHD and depression [35,39], and one conducted multiple explorations that were mixed but largely null [37]. The only two studies that used a clinical depression outcome found no increased risk of depression for low 25OHD [34,40]. The study that accounted for all key confounders found no association between PPD and continuous or deficient 25OHD in for the most part, although women with deficient 25OHD who did not receive omega-3 supplements did have an increased risk of PPD at one of the two timepoints assessed [37].

## 4. Discussion

This is the first systematic review of the evidence on vitamin D and PPD from both RCTs and observational studies. We found the totality of the evidence poor and inconclusive. There were only two RCTs of vitamin D supplementation, and although both claimed a benefit of vitamin D for depressive symptoms, sample sizes were insufficient to provide adequate power and intervention periods were short [29,30]. The observational studies were equally inconclusive, with some reporting a link between vitamin D and depressive symptoms in the antenatal [39,41,42,43] or postpartum period [32,33,35,36,37,39,49], and many detecting no association [29,31,40,46,47,48]. Numerous methodological limitations included insufficient samples, inappropriate exposure or outcome measures, or lack of adjustment for confounders, particularly season, which is known to be a major determinant of 25OHD status [57]. The case of vitamin D and postpartum depression highlights a missed opportunity, in which the limited contradictory observational studies were insufficient to justify including a measure of depressive symptoms in over 30 published RCTs of prenatal vitamin D supplementation [58].

Although there is biological plausibility for a role of 25OHD in the development of depression, the current literature base has been unable to demonstrate this. Our mixed, inconclusive results align with other reviews of vitamin D and depression during or outside of the perinatal period [9,23,24,25,26,59,60,61,62,63,64,65,66,67,68,69,70]. All called for further, high quality research to provide conclusive evidence, and several recommended that vitamin D supplementation only be considered for individuals with deficient 25OHD status [11,18,23,24,25,26,27,68,69,70].

Given that measures of depression in the perinatal period, such as the short self-completed EPDS questionnaire, are simple to administer, we recommend that depression is included as an outcome in current incomplete RCT’s of prenatal vitamin D as well as future trials. Future observational studies exploring associations between 25OHD status should use LC-MS/MS and standardize 25OHD for the season, as well as assessing perinatal depression with a measure suitable for use in the perinatal period, whilst adjusting for the minimum key confounders of age, education (and/or other indicators of socio-economic status), history of depression, and multivitamin or vitamin D supplement use, and ideally, smoking, body mass index, and ethnicity. Further, it would be prudent for any vitamin D RCTs to specifically target women with low or deficient 25OHD rather than a sufficient sample who is unlikely to benefit from additional vitamin D exposure. We advise that future studies of perinatal vitamin D and depressive symptoms strongly consider the PRISMA (Supplementary Materials) and CONSORT statements when designing and conducting studies.

## 5. Conclusions

The currently available evidence from RCTs, cohort studies, and case-control studies are insufficient to establish a role of vitamin D in the pathophysiology, prevention, or treatment of depression in the perinatal period.

## Figures and Tables

**Table 1:** Randomized controlled trials of vitamin D supplementation in the antenatal and postpartum period and depression.

Author, Year Sample, Design, Setting	Vitamin D Intervention	PPD Measure and Definition	Results and Limitations
Vaziri, 2016 [29] Iran *n* = 169, healthy women >18, singleton pregnancy 26–28 wks gestation Excluded women with a history of mental illness, EPDS > 13, substance abuse, pregnancy complications Design: single-blinded RCT Primary outcome: NR Recruited at prenatal hospital	Duration: 26–28 wks gestation until birth Trt *n* = 78; 50 µg vitamin D3/day Ctrl *n* = 75; placebo	EPDS (Iranian version, via interview) at 38–40 wks gestation EPDS (Iranian version, via interview) at 4 and 8 wks PP Depression = EPDS > 13	*N* = 136 (80%) 38–40 wks: Trt depression < Ctrl 4 wks PP: Trt depression < Ctrl 8 wks PP: Trt depression < Ctrl Limitations: small sample, analyses per protocol not intention to treat (actively excluded women who took supplements irregularly or ceased supplements), EPDS conducted via interview, blinding of staff and participants unclear, randomization methods unclear, single-blinded study only, many women taking daily prenatal multivitamin with 200–400 IU vitamin D, participants apparently unlikely to develop depression at enrollment, no primary outcome specified
Amini, 2020 [30] Iran, *n* = 81 women aged 18–45 yrs, EPDS > 12 Excluded BMI ≥ 35, 25OHD status > 75 nmol/L, previous history of depression or other mental disorder, antidepressant use Design: double-blinded RCT Primary outcome: PPD and serum 25OHD Recruited at psychiatric outpatient clinic	Duration: 8 wks PP (exact timing of intervention commencement NR) Trt1 *n* = 27; oral 1250 µg vitamin D3/fortnight + 500 mg calcium carbonate/day Trt2 *n* = 27; oral 1250 µg vitamin D3/fortnight + placebo/day Ctrl *n* = 27; placebo	EPDS (Iranian version, via interview) at end of intervention |PPD = EPDS ≥ 12	*N* = 76 (94%) Trt1 and Trt2 PPD < Ctrl Limitations: small sample, EPDS conducted via interview, calcium supplement group (Trt1) combined with vitamin D only group (Trt2) to compare to controls, randomization methods unclear

BMI: body mass index, Ctrl: control, EPDS: Edinburgh Postnatal Depression Scale, IU: International units, NR: not reported, PP: Post-partum, PPD: postpartum depression, Trt: treatment, and Wks = weeks.

**Table 2:** Observational studies of vitamin D and symptoms of depression during pregnancy and postpartum.

Author, Year Setting, Sample	Vitamin D Measure and Classification	PPD Measure and Definition Confounders	Results and Limitations
Murphy, 2010 [33] U.S.A.: prospective cohort (original study design NR) *N* = NR, women taking 20, 2400 or 6400 IU vitamin D/day Excluded births <35 wks gestation, pre-existing diabetes or a multiple birth Recruitment setting NR	Sample: serum 25OHD monthly from 4–6 wks PP to 7 mo PP Sample analysis: Radioimmunoassay Sufficient: ≥80 nmol/L Insufficient: 50–≤80 nmol/L Deficient: ≤50 nmol/L	EPDS (English and Spanish versions) measured monthly from 4–6 wks PP to 7 mo PP PPD = EPDS > 9 Confounders: season, age, education, infant sex, marital status, insurance status, infant feeding method, vitamin D dose, planned pregnancy	*N* = 97 (%NR) Continuous: NA Categorical: Low PP serum 25OHD increased risk of PPD during the first 7 mo PP Limitations: small likely underpowered sample, women provided with vitamin D supplements at 3 doses and sample appeared to be drawn from a dose-response trial, did not include confounders’ history of depression
Cassidy–Bushrow, 2012 [43] U.S.A.: cohort (original study design NR) *N* = 203, African-American women who spoke and read English and were in their second trimester Excluded illicit drug use, psychiatric illness Hospital obstetric clinic recruitment	Sample: serum 25OHD at first trimester (mean 9.5 wks gestation) Sample analysis: chemiluminescence immunoassay Sufficient: >50 nmol/L Insufficient: 30-50 nmol/L Deficient: <30 nmol/L	CES-D during pregnancy (timing NR) Depression = CES-D ≥ 16 Confounders: season, education, marital status, days between exposure and outcome	*N* = 178 (88%NR) Continuous: low 25OHD status increased risk of depression Categorical: deficient 25OHD increased risk of depression Limitations: small likely underpowered sample, women provided with vitamin D supplements with higher doses prescribed after exposure measure, did not include confounders’ history of depression, age, or supplement use
Brandenbarg, 2012 [41] Netherland: prospective cohort (pregnancy and child cohort) *N* = 8266, inclusive of race and language spoken First antenatal clinic visit recruitment	Sample: serum 25OHD at early-pregnancy (median 13 wks gestation) Sample analysis: enzyme immunoassay Sufficient: ≥50 nmol/L Insufficient: 30–49.9 nmol/L Deficient: ≤29.9 nmol/L	CES-D (Dutch, English, Arabic and Turkish version) during pregnancy at 16 wks gestation Depression = CES-D ≥ 16 Confounders: season, age, parity, ethnicity, BMI, smoking, drinking, planned pregnancy, education, cohabitation status, employment status	*N* = 4101 (50%) Continuous: low 25OHD status increased risk of depression Categorical: deficient 25OHD increased risk of depression Limitations: did not include confounder history of depression
Nielsen, 2013 [34] Denmark: case-control (nestled in a birth cohort of 91,000 women) *N* = 605 with PPD (filled prescription for antidepressant) *N* = 875 without PPD (no prescription, matched for age and year of recruitment) Singleton pregnancy with live-born infant, excluded women with previous registered mental illness or anti-depressant use in the year prior to giving birthRecruitment setting NR	Sample: serum 25OHD at mid-pregnancy (25 wks gestation) Sample analysis: LC-MS/MS Exposure categorized as <15 nmol/L, 15–24 nmol/L, 25–49 nmol/L, 50–70 nmol/L, 80–99 nmol/L, ≥100 nmol/L	Danish Register of Medicinal Product Statistics at 12 mo PP PPD = prescription for any anti-depressant medication Confounders: season, week of gestation at exposure measure, age, parity, smoking, socioeconomic status, BMI, physical activity, social support, multivitamin supplement use	*N* = 1480 (%NA) Continuous: NA Categorical: No increased risk of PPD at lower levels, increased risk if 25OHD < 80 nmol/L Limitations: crude measure of depression (severe depression only), did not include confounder history of depression
Robinson, 2014 [35] Australia: prospective cohort (pregnancy cohort) *N* = 2900, Caucasian women Maternity hospital recruitment	Sample: serum 25OHD at early-pregnancy (18 wks gestation) Sample analysis: LC-MS/MS Quartile 1: <47 nmol/L Quartile 2: 47–58 nmol/L Quartile 3: 59–70 nmol/L Quartile 4: >70 nmol/L	EPDS (English, revised to 6 items only) at 3 days PP PPD = EPDS > 6 Confounders: season, age, education, family income, BMI, smoking, drinking, hypertensive disease, infant sex, child admission to special care nursery, birthweight	*N* = 706 (24%) Continuous: NA Categorical: Low serum 25OHD increased risk of PPD at 3 days Limitations: EPDS used within 1 wk of birth (instead of recommended >14 days), used an unvalidated abbreviated version of EPDS, did not include confounders’ history of depression or supplement use
Fu, 2014 [32] China: prospective cohort (PPD cohort) *N* = 323, women who gave birth to a full-term, singleton Excluded if psychiatric care during pregnancy City hospital recruitment at birth	Sample: serum 25OHD at 24–48 h after delivery Sample analysis: E601 modular analyzer Sufficient: >75 nmol/L Insufficient: 50–75 nmol/L Deficient: <50 nmol/L	EPDS (Chinese version) at 3 mo PP PPD = EPDS ≥ 12 Confounders: age, breastfeeding, stressful life events, education, family income, partner support, planned pregnancy, mode of delivery, previous psychiatric contact	*N* = 213 (66%) Continuous: 25OHD status higher in women without PPD Categorical: Low 25OHD more likely to have PPD Limitations: small likely underpowered sample, cohort analyzed as case-control, did not include confounders season, or supplement use
Huang, 2014 [31] U.S.A: cohort (for pregnancy migraine study) *N* = 500, women who sought prenatal care prior to 20 wks gestation, spoke English, >18 years Recruitment setting NR	Sample: serum 25OHD at early-pregnancy (mean 15.4 wks gestation) Sample analysis: LC-MS/MS Sufficient: ≥83 nmol/L Insufficient: 51–≤83 nmol/L Deficient: ≤50 nmol/L	DASS-21 and PHQ-9 in early pregnancy (mean 15.4 wks gestation) Depression = DASS ≥ 14 =PHQ-9 ≥ 19 Confounders: season, gestation of exposure, age, BMI, smoking, race, education, marital status	*N* = 498 (99.6%) Continuous: No association Categorical: No association Limitations: moderate sample size, suboptimal outcome measure, did not include confounders history of depression or supplement use
Gur, 2014 [36] Turkey: prospective cohort (community cohort study) *N* = 687, Normal pregnancy and delivery, Excluded if risk of PPD, or complications with birth or neonate University hospital recruitment	Sample: serum 25OHD at mid-pregnancy (24–28 wks gestation) Sample analysis: enzyme-linked immunosorbent assay Sufficient: >50 nmol/L Mildly deficient: 26–≤50 nmol/L Severely deficient: ≤25 nmol/L	EPDS (Turkish version) at 1 wk, 6 wks and 6 mo PP PPD = EPDS ≥ 12 Confounders: none reported	*N* = 179 (26%) Continuous: women with PPD had lower 25OHD Categorical: Low serum 25OHD increased risk of PPD at 1 and 6 wks and 6 mo Limitations: small likely underpowered sample, cohort analyzed as case-control, women provided with vitamin D supplements, EPDS used within 1 wk of birth (instead of recommended >14 days), EPDS completed via interview instead of self-completed, did not appear to account for any confounders
Gould, 2015 [37] Australia: Prospective, enrolled at ~20 wk gestation (for pregnancy omega-3 trial) *N* = 2399, singleton pregnancy, healthy women, <20 wks gestation Excluded illicit drug use Hospital antenatal recruitment	Sample: cord blood 25OHD at birthSample analysis: LC-MS/MS Sufficient: >50 nmol/L Insufficient: 25–50 nmol/L Deficient: <25 nmol/L	EPDS (English version) at 6 wks and 6 mo PP PPD = EPDS > 12 Confounders: season, social support, age, race, parity, BMI, education, history of depression, prenatal supplement use, prenatal smoking	*N* = 1040 (43%) Continuous: No association (6 wks or 6 mo) Categorical: Deficiency at 6 wks increased risk of PPD (in placebo group). No increased risk in pmega-3 group at 6 wks, and no risk at 6 mo (omega-3 or placebo group) Limitations: possible interference of omega-3 intervention
Miyake, 2015 [42] Japan: cross-sectional cohort (maternal-child health cohort) *N* = 1757 women 5–39 wks gestation Obstetric hospital recruitment	Sample: vitamin D intake at 5–39 wks gestation Sample analysis: diet history questionnaire	CES-D (Japanese version) during pregnancy at 5–39 wks gestation Depression = CES-D ≥ 16 Confounders: age, gestation, region, parity, family structure, history of depression, smoking, occupation, family income, education, BMI, intake of saturated fatty acids and omega-3 fatty acids	*N* = 1745 (99%) Continuous: NA Categorical: Higher dietary vitamin D intake associated with lower risk of depression Limitations: original cohort used to show increased seafood associated with less depression but did not consider this in analyses, did not asses 25OHD status, analyzed dietary intake (mainly as fish and eggs, vitamin D supplements not captured) rather than measuring sun exposure which is main source of vitamin D, dietary patterns likely varied within the 34 week period of diet assessment due to morning sickness in early pregnancy and increased intake in late pregnancy, did not include confounders’ season or supplement use
Miyake, 2016 [47] Japan: cross-sectional cohort (maternal-child health cohort, from [42]) *N* = 1757 women 5-39 wks gestation Obstetric hospital recruitment	Sample: vitamin D dietary intake at 5–39 wks gestation Sample analysis: diet history questionnaire	EPDS at 3–4 mo PP PPD = EPDS ≥ 9 Confounders: age, gestation, region, parity, family structure, history of depression, occupation, education, BMI, smoking, cesarean delivery, infant sex, birth weight, total energy intake	*N* = 1319 (75%) Continuous: NA Categorical: No association of low dietary vitamin D with PPD Limitations: as above, inconsistent confounders to above
Accortt, 2016 [48] U.S.A.: prospective cohort (cohort from [43]) *N* = 203, African-American women who spoke and read and were in their second trimester Excluded illicit drug use, psychiatric illness Hospital obstetric clinic recruitment	Sample: serum 25OHD at first trimester (mean 9.5 wks gestation) Sample analysis: chemiluminescence immunoassay Sufficient: NR Insufficient: NR Deficient: ≤25 nmol/L, ≤37.5 nmol/L, and ≤75 nmol/L	EPDS at 4-6 wks PP PPD = not defined, used continuous EPDS score Confounders: season, age, education, marital status, history of depression, BMI	*N* = 91 (45%) Continuous: No association Categorical: No increased risk from deficiency Limitations: designed for exploring combined effect of vitamin D and inflammatory biomarkers, small likely underpowered sample, women provided with vitamin D supplements, did not include confounder supplement use
Gunduz, 2016 [46] Turkey: prospective cohort *N* = 91, women with full-term singleton, took 500 IU vitamin D throughout pregnancy Excluded mental health problems University maternity clinic recruitment	Sample: serum 25OHD at 36 wks gestation Sample analysis: high performance liquid chromatography Sufficient: NA Insufficient: <32 nmol/L Deficient: <20 nmol/L	EPDS at 6 wks PP PPD = EPDS ≥ 10 Confounders: infant crying, relationship with the partner, infant weight gain, feeding type	*N* = 87 (94%) Continuous: No association Categorical: No increased risk from deficiency Limitations: small likely underpowered sample, women provided with vitamin D supplements, did not include any key confounders
Vaziri, 2016 [29] Iran: cross sectional (vitamin D RCT) *N* = 169, healthy women >18, singleton pregnancy 26–28 wks gestation, living with husband Excluded history of mental illness, EPDS > 13, substance abuse, pregnancy complications Prenatal hospital recruitment	Sample: serum 25OHD 26–28 at wks gestation Sample analysis: chemiluminescence immunoassay	EPDS (Iranian version, via interview) at 26–28 ks gestation Confounders: none reported	*N* = 136 (80%) Continuous: No association Categorical: NA Limitations: small likely underpowered sample, EPDS completed via interview instead of self-completed, did not appear to account for any confounders
Williams, 2016 [40] U.S.A.: prospective cohort (for pregnancy omega-3 trial to prevent depression) *N* = 126, pregnant women at risk of depression, with singleton pregnancy 12–20 wks gestation Excluded current depression or antidepressant medication use, substance abuse Prenatal clinic recruitment	Sample: serum 25OHD at 12–20 wks and 34–36 wks Sample analysis: radioimmunoassay Sufficient: ≥50 nmol/L Deficient: <50 nmol/L	BDI and MINI at 10–20 wks, 26–28 wks and 34–36 wks gestation, and 6–8 PP PPD = NR Confounders: season, age, smoking, BMI, initiation of antidepressants, omega-3 fatty acid status	*N* = 105 (83%) Continuous: No association Categorical: No increased risk from deficiency Limitations: small likely underpowered sample, women provided with vitamin D supplements, did not include confounders’ education, supplement use or history of depression
Abedi, 2018 [45] Iran: case-control study *N* = 60 with PPD (definition NR) *N* = 60 without PPD (definition NR, matched to age and whether taking vitamin D supplements) Women 6–8 wks PP Public health clinic recruitment	Sample: serum 25OHD at PP (timing NR) Sample analysis: enzyme-linked immunosorbent assay Sufficient: >75 nmol/L Deficient: <50 nmol/L	BDI (Iranian version, via interview) at PP (timing NR) Confounders: age, education, husbands’ education, income, BMI	*N* = 120 Continuous: 25OHD lower among cases Categorical: deficiency more likely in cases Limitations: small likely underpowered sample, cases and controls not defined, did not include confounders’ season, supplement use or history of depression
Lamb, 2018 [39] U.S.A.: prospective cohort *N* = 126 women <25 wks gestation Excluded pre-existing mental conditionObstetric clinic recruitment	Sample: serum 25OHD at early pregnancy (mean 14 wks gestation), and at delivery, and at 6 wks PP Sample: cord blood at birth Sample analysis: LC-MS/MS Sufficient: >75 nmol/L Insufficient: 50-75 nmol/L Deficient: ≤50 nmol/L	EPDS at 14 wks gestation, 32 wks gestation, and at 10 wks PP PPD = EPDS ≥ 10 Confounders: history of depression, supplement use	*N* = 125 (99%) Continuous: 25OHD status in maternal and cord blood associated with depression Categorical: maternal deficiency associated with increased risk of depression Cord blood: NR Limitations: small likely underpowered sample did not include confounders’ season, age, or education
Jani, 2020 [38] Australia: retrospective cross-sectional cohort *N* = 17,132, all women who gave birth in the target region during the study period Excluded multiple pregnancies and missing key data Recruited via accessing medical records of births in study period	Sample: serum 25OHD at ~14 wks gestation Sample analysis: NR Sufficient: >50 nmo/L Deficient: ≤50 nmol/L	EPDS at 12-14 wks gestation Depression = EPDS ≥ 13 Confounders: season, age, parity, marital status, smoking, birthweight, maternal country of birth, employment status, domestic violence, hypertension during pregnancy	*N* = 13,805 (81%) Continuous: NA Categorical: maternal deficiency associated with increased risk of depression Limitations: unclear measure of 25OHD, did not include confounders’ supplement use, or history of depression
Accortt, 2021 [49] U.S.A.: prospective cohort (analyzed as case vs. control) *N* = NR, singleton pregnancy, <20 wks gestation Prenatal clinic recruitment	Sample: plasma 25OHD at 18–21 wks gestation Sample analysis: LC-MS/MS in multiple reaction monitoring mode—“vitamin D metabolites” Vitamin D ration ratio of 24,25OHD and 25OHD	CES-D at 6–10 wks PP PPD = CES-D ≥ 16 Confounders: BMI, age, smoking, race, prenatal depression	*N* = 89 (56% of the 160 with vitamin D status) Continuous: women with PPD had lower vitamin D ratio Categorical: NA Limitations: small likely underpowered sample, cohort analyzed as case-control, analyzed vitamin D metabolite ratio (rather than 25OHD status), did not include confounders’ season or supplement use
Pillai, 2021 [44] India: cross-sectional case-control *N* = 330 cases (EPDS ≥ 10) *N* = 330 controls (EPDS < 10), matched for age and BMI Excluded women with transient mood changes, postpartum blues, pre-existing depressive symptoms that commenced prior to birth Postpartum clinic recruitment	Sample: serum 25OHD at 6 wks PPSample analysis: enzyme-linked immunosorbent assay Sufficient: >75 nmol/L Deficient: ≤75 nmol/L	EPDS (English or Tamil translation) at 6 wks PP Confounders: age, BMI, socioeconomic status, marriage satisfaction, adverse events during pregnancy, fear of labor, prenatal medical conditions, kangaroo care, child care stress	*N* = 660 (%NA) Continuous: lower 25OHD status in controls than casesCategorical: cases more likely to be deficient than controls Limitations: women provided with vitamin D supplements, did not include confounders’ season, education, history of depression, or supplement use

25OHD: 25-hydroxyvitamin D (vitamin D), BDI: Beck Depression Inventory, CES-D: Center for Epidemiological Studies Depression scale, DASS: Depression, Anxiety and Stress Scale, EPDS: Edinburgh Postnatal Depression Scale, LC-MS/MS = Liquid chromatography-tandem mass spectroscopy, MINI = Mini International Neuropsychiatric Interview, mo = months, NA: not available, NR: not reported, PHQ-9: Patient Health Questionnaire Depression Module, PP: Post-partum, PPD: postpartum depression, and wks = weeks.

## Data Availability

Not applicable.

## Supplementary Materials

The following supporting information can be downloaded at: https://www.mdpi.com/article/10.3390/nu14112300/s1, PRISMA 2020 Checklist.
